# A marker chromosome in post-transplant bone marrow

**DOI:** 10.1186/s13039-016-0250-z

**Published:** 2016-06-01

**Authors:** Laura Morsberger, Kerry Powell, Yi Ning

**Affiliations:** Department of Pathology, Johns Hopkins University School of Medicine, 600 N. Wolfe Street, Baltimore, MD 21287 USA

**Keywords:** Marker chromosomes, Isodicentric chromosomes, FISH, Bone marrow transplantation

## Abstract

Detection of small supernumerary marker chromosomes in karyotype analysis represents a diagnostic challenge. While such markers are usually detected during cytogenetic studies of constitutional chromosome abnormalities, they have also been found in specimens submitted from patients with acquired malignancies. We report here the detection of a marker chromosome in a bone marrow specimen from a patient who received a bone marrow transplantation. We discuss the importance of proper characterization and interpretation of marker chromosomes in clinical practice.

## Letter to Editor

We read with interest of an article recently published in Molecular Cytogenetics by Wang et al. [1] entitled “Copy number changes and methylation patterns in an isodicentric and a ring chromosome of 15q11-13: report of two cases and review of literature.” We would like to extend the discussion of marker chromosomes and report our finding of a small supernumerary marker chromosome in a patient who received a bone marrow transplantation.

Marker chromosomes and ring chromosomes are structurally abnormal chromosomes of unknown origin. Small supernumerary marker chromosomes frequently represent a challenge in cytogenetic diagnosis. Accurate characterization of such marker chromosomes is of diagnostic and management significance. In a recent book, “Small supernumerary marker chromosomes (sSMC): a guide for human geneticists and clinicians,” Dr. Thomas Liehr [2] provided extensive review and discussion on the topic, and categorized patients with sSMC into four groups: 1) prenatally studied ones (with and without sonographic abnormalities), 2) postnatally examined adults with fertility problems, 3) children and adults with unclear mental retardation, developmental delay, and/or dysmorphism, and 4) patients in which sSMC can be a secondary finding when cytogenetic analysis is done for other reasons. While the discussion of marker chromosomes usually involves cytogenetic study of constitutional chromosome abnormalities, either during prenatal diagnosis or delineating pathogenesis of postnatal cases, marker chromosomes of unknown origin have also been detected during karyotype analysis of specimens from patients with acquired malignancies. Proper characterization and interpretation of observed marker chromosomes is important in cytogenetic diagnosis, which can also guide clinical management.

**Fig. 1:**
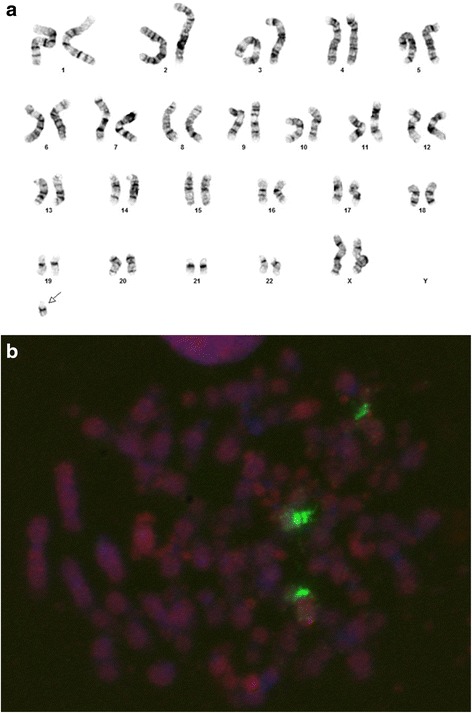
Detection of a marker chromosome in a post-transplant bone marrow specimen. **a** A marker chromosome of unknown origin, indicated by an arrow, was observed following a sex-mismatched bone marrow transplantation. **b** FISH using a chromosome 15 centromere probe, labelled in green, showed two copies of chromosome 15 as well as an isodicentric chromosome 15 in a metaphase cell

**Fig. 2:**
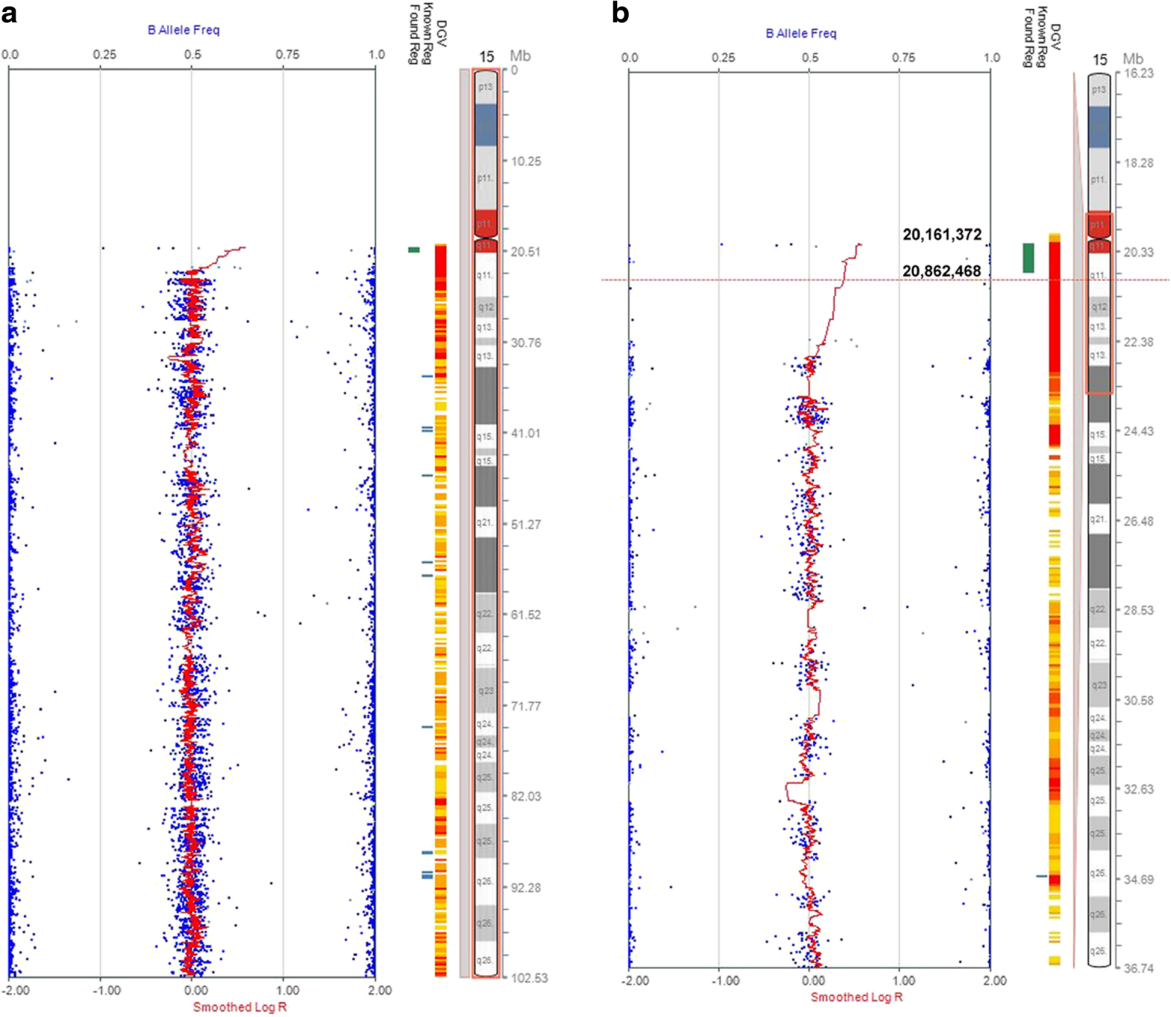
SNP array profile of chromosome 15 from the transplantation donor. **a**) The profile of entire chromosome 15 from 0 to 102.53 Mb, as indicated at right of chromosome 15 ideogram. **b**) Zoom-in region of chromosome 15 from 16.23 to 36.74 Mb, as indicated at right of chromosome 15 ideogram. The Illumina SNP array platform indicated a gain from 20.16 to 20.86 (0.7 Mb), shown in green at left of chromosome 15 ideogram under “found region.” However, this is within the polymorphic, non-pathogenic region adjacent to the centromere of chromosome 15, consistent with the normal phenotype of the healthy transplantation donor

Our patient was a 71-year-old male with history of myelodysplastic/myeloproliferative disorder. At the time of diagnosis, he presented a male karyotype with loss of a copy of chromosome 7 and gain of an extra copy of chromosome 19. The karyotype was described as 46,XY,-7,+19[20]. This patient received a bone marrow transplantation, with his daughter as the transplantation donor. Karyotype analysis performed following bone marrow transplantation revealed a female karyotype that included an extra small marker chromosome with acrocentric appearance (Fig. 1a). Considering that about 50 % of small marker chromosomes are derivatives of chromosome 15, and such markers with the presence of a limited amount of euchromatin are likely associated with a normal phenotype [3], we performed fluorescence in situ hybridization (FISH) using a chromosome 15 centromere probe to characterize this marker. As shown in Fig. 1b, the marker appeared to be an isodicentric chromosome 15. We further confirmed that the marker did not contain the Prader-Willi/Angelman syndrome critical region. We reported the observed karyotype as //47,XX,+psu idic(15)(q11.2)[20], and described that the marker likely represented the constitutional karyotype of the donor. The presence of female sex chromosomes in all analyzed cells was indicative of donor hematopoiesis. Post-transplant chimerism study confirmed 100 % donor DNA in patient’s bone marrow. Findings from single nucleotide polymorphism (SNP) microarray analysis of donor chromosome 15 are shown in Fig. 2. Consistent with normal phenotype of the donor, no pathogenic copy number change or uniparental disomy is evident.

The sex mismatch bone marrow transplantation in this case facilitated the recognition of the marker to be donor cell in origin. We report this case here to demonstrate that incidental findings are not uncommon in clinical settings, and that the diagnostic laboratories should provide informative interpretation to help the understanding of usual findings.

In the study by Wang et al. [1], the authors combined karyotyping, FISH, microarray, and methylation-specific multiplex ligation-dependent probe amplification approaches to effectively determine the origin, size, and epigenetic pattern of unknown marker chromosomes in two diagnostic specimens. Evaluation of the methylation pattern of markers and rings originating from chromosome 15 is of clinical significance because the Prader-Willi/Angelman syndrome critical region of 15q11-q13 contains many imprinting genes and shows the parental-origin effects [4, 5]. We performed FISH following karyotype analysis to determine that the marker is an isodicentric chromosome 15, because this was an incidental observation in a post-transplant bone marrow, the marker seemed to be from donor cells and the donor is a healthy individual.

We report this case to raise the awareness that benign constitutional chromosome abnormalities can be occasionally seen in specimens submitted for cytogenetic study of acquired malignancies. Application of molecular cytogenetic techniques, such as multicolor FISH [6, 7] and microarray analysis [1, 8], would enable us to accurately characterize marker or mosaic marker chromosomes in patient specimens and contribute to improved patient care.
